# User-documented food consumption data from publicly available apps: an analysis of opportunities and challenges for nutrition research

**DOI:** 10.1186/s12937-018-0366-6

**Published:** 2018-06-09

**Authors:** Marcus Maringer, Pieter van’t Veer, Naomi Klepacz, Muriel C. D. Verain, Anne Normann, Suzanne Ekman, Lada Timotijevic, Monique M. Raats, Anouk Geelen

**Affiliations:** 1 Division of Human Nutrition, Wageningen University & Research, Wageningen, The Netherlands; 2 Food, Consumer Behaviour and Health Research Centre, University of Surrey, Guildford, Surrey, United Kingdom; 3 Wageningen Economic Research, Wageningen University & Research, Wageningen, The Netherlands; 4 Division of Bioscience and Materials, Agrifood and Bioscience, RISE Research Institutes of Sweden, Gothenburg, Sweden

**Keywords:** Food consumption data, Dietary intake assessment, Diet apps, User-documented data, Contextual data, Technological innovations, Data management, Legal and ethical governance, Research infrastructure

## Abstract

**Background:**

The need for a better understanding of food consumption behaviour within its behavioural context has sparked the interest of nutrition researchers for user-documented food consumption data collected outside the research context using publicly available nutrition apps. The study aims to characterize the scientific, technical, legal and ethical features of this data in order to identify the opportunities and challenges associated with using this data for nutrition research.

**Method:**

A search for apps collecting food consumption data was conducted in October 2016 against UK Google Play and iTunes storefronts. 176 apps were selected based on user ratings and English language support. Publicly available information from the app stores and app-related websites was investigated and relevant data extracted and summarized. Our focus was on characteristics related to scientific relevance, data management and legal and ethical governance of user-documented food consumption data.

**Results:**

Food diaries are the most common form of data collection, allowing for multiple inputs including generic food items, packaged products, or images. Standards and procedures for compiling food databases used for estimating energy and nutrient intakes remain largely undisclosed. Food consumption data is interlinked with various types of contextual data related to behavioural motivation, physical activity, health, and fitness. While exchange of data between apps is common practise, the majority of apps lack technical documentation regarding data export. There is a similar lack of documentation regarding the implemented terms of use and privacy policies. While users are usually the owners of their data, vendors are granted irrevocable and royalty free licenses to commercially exploit the data.

**Conclusion:**

Due to its magnitude, diversity, and interconnectedness, user-documented food consumption data offers promising opportunities for a better understanding of habitual food consumption behaviour and its determinants. Non-standardized or non-documented food data compilation procedures, data exchange protocols and formats, terms of use and privacy statements, however, limit possibilities to integrate, process and share user-documented food consumption data. An ongoing research effort is required, to keep pace with the technical advancements of food consumption apps, their evolving data networks and the legal and ethical regulations related to protecting app users and their personal data.

**Electronic supplementary material:**

The online version of this article (10.1186/s12937-018-0366-6) contains supplementary material, which is available to authorized users.

## Background

With the widespread use of mobile phones and tablets, there has been an increase in the number of software applications that record and aim to improve people’s food consumption behaviour [1–4]. The need for more suitable and effective methods for measuring, understanding and influencing food consumption behaviours has sparked interest amongst behavioural and nutrition researchers for these digital solutions. Smartphones and their implemented technologies such as barcode scanners, image processors, microphones, databases, and wireless network interfaces have the potential to enhance the accuracy and efficiency of data collection and reduce the costs and inconvenience of assessing diets in real time [1, 5–7]. Previous research provides vital insights regarding features and functionalities of publicly available food consumption apps [1, 2, 8], their effectiveness for weight loss interventions and improving nutrition related behaviours [4, 6, 9–11], the quality of the provided information and implemented behavioural change techniques [12–16], user adherence [6, 10, 17], app usability and perceived usefulness [2, 18].

Accompanied by the growing interest in new and efficient technologies for recording and improving people’s food consumption behaviours, there is growing interest in the collection and investigation of the large stream of food consumption data, which is generated by the vast amount of users of these technologies. Investigating such *user-documented* food consumption data, which is data that has already been collected by users of apps (e.g., for self-monitoring purposes), is in itself highly efficient because such secondary data usage reduces the costs for collecting data and reduces the burden on respondents [19, 20]. More importantly, food-related consumer behaviours are most often studied in isolation, in short time frames and in a relatively limited social and physical context [21]. Every day, users of diet apps generate “big data” - large volumes of information, that offer detailed descriptions of food consumptions, including time and place (e.g., using Global Positioning Systems; GPS). If these data-rich sources could be linked and analyzed, they have the potential to contribute greatly towards answering key questions regarding food and health (e.g., obesity, cardiovascular disease) and to a better understanding of food consumption behaviour including its drivers and barriers [22]. In order to advance health and nutrition research, the European Union (EU) funded RICHFIELDS project (http://www.richfields.eu) aims to design an EU-wide research infrastructure (RI) and distributed open access data platform for the collection, integration, and sharing of food consumption data from various sources including the increasing stream of food consumption data documented by users of nutrition apps.

The use of user-documented data, however, creates new challenges, which go beyond the type and quality of implemented app features. These challenges involve procedures of finding and retrieving relevant data, the methods and purposes of data collection, informed consent, confidentiality, and data ownership [20, 23]. It was our aim to investigate the characteristics and qualities of user-documented food consumption data in order to learn more about its *scientific relevance* in regarding its potential for estimating habitual food intake and for providing a better understanding of the determinants of food consumption behaviours. In addition, we focused on characteristics relevant for *data management* practices including data access and data integration. This information is important for implementing data processing strategies that rely on effective and reliable data exchange protocols. Finally, we focused on characteristics of the data relevant to its *legal and ethical governance*. The rights, obligations, and expectations regarding data usage are important since failure to adhere to these regulations might compromise data integrity [24]. In sum, in the present research we focused on evaluating characteristics of apps, which relate to the secondary usage of data generated by regular “users” of publicly available apps, which we refer to as user-documented data. Our aim was to provide an overview of important scientific, technical, legal and ethical aspects of user-documented food consumption data that should inform researchers about the opportunities and challenges associated with collecting and investigating this type of data for nutrition research.

## Methods

### App identification

**Fig. 1:**
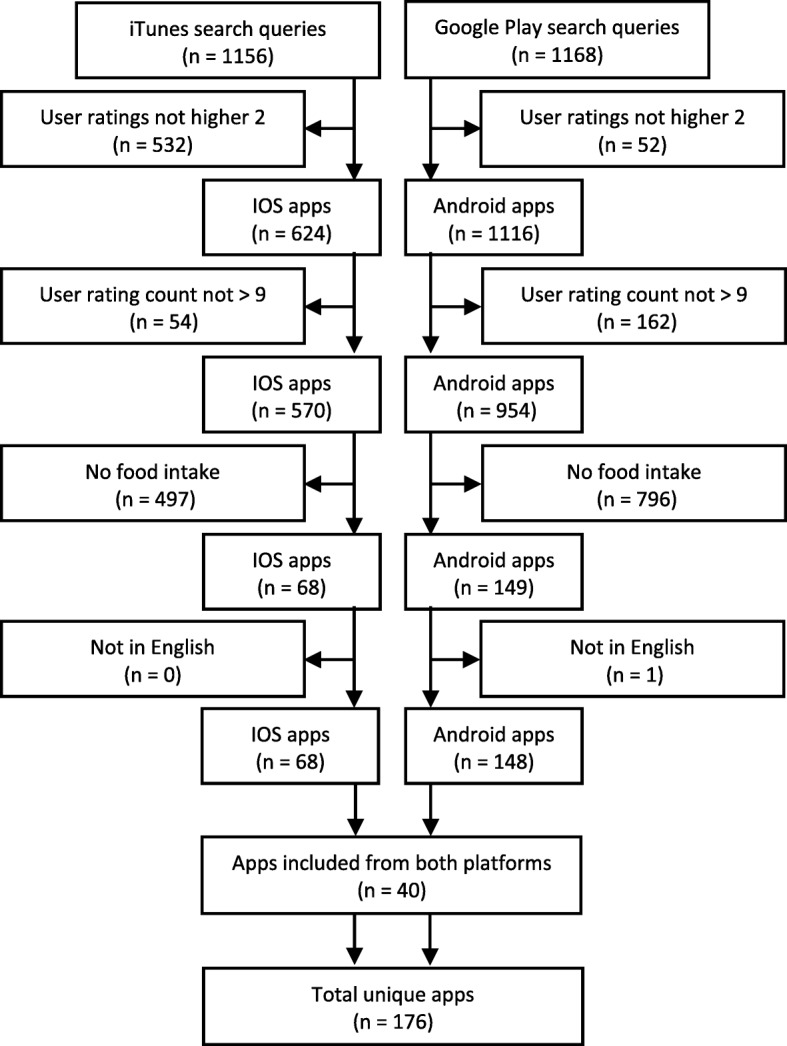
Flow diagram of app search and selection

The iTunes and Google Play stores were searched between 15 and 23 October 2016 in order to identify apps which allow the user to collect food consumption data. A set of search terms created by Franco et al. in their review of popular nutrition apps [1] were adopted. Search terms included: calorie(s), diet, diet tracker, dietician, dietitian, eating, fit, fitness, food, food diary, food tracker, health, lose weight, nutrition, nutritionist, weight, weight loss, weight management, weight watcher, and ww calculator. Automated data collection techniques were used for both apps stores. Each search term was queried separately without combining individual search terms. For the iTunes store, app data was queried from the public iTunes Search application programming interface (API) [25]. For the Google Play Store, app data was extracted by a web data crawling software [26]. The open source Nodejs module itunes-search^1^ (version 1.0.1) was used to collect data from the iTunes search API, and the open source Nodejs module google-play-scraper^2^ (version 0.2.1) was used to collect data from the Google Play Store. For more detailed documentation regarding the usage of these two Nodejs modules for conducting searches against the Google Play Store and the iTunes search API, please refer to the documentation and examples provided in their public repositories. Our aim was to limit the number of apps to only the most relevant with an already established user-base and a certain degree of app quality. To limit the apps identified, the modules were configured to retrieve only the first 100 applications for each search term. Search results were further limited by means of app user ratings. Both iTunes and Google Play stores provide app users with a function to rate their liking of the apps on a 5-point scale. Apps from the returned searches that had a mean user rating of more than 2 (based on a minimum of 10 user ratings) were retained for use in this study. To ensure the retrieval of English language apps, United Kingdom (UK) storefronts were searched only. No affiliate account or token was used at the iTunes Search API. This search strategy resulted in the collection of 176 unique apps (see Fig. 1).

### User-documented data characterization

#### Information sources

Descriptions of apps and services were taken from publicly available information for each app published by the app vendors. This information included the technical details, app descriptions and screenshots provided in the respective app stores (iTunes and Google Play Store) and, where available, feature and service descriptions, documentation, and frequently asked questions on associated homepages. Terms of use and privacy statements were reviewed to identify information relevant to legal and ethical governance.

#### Data characteristics

A list of characteristics related to user-documented data was generated for the extraction of information from the defined information sources. The criterion for inclusion of a characteristic was based on whether information regarding the characteristic could be expected to be publicly available, without the need to install and use the app. Specifically, there is a vast array of quality criteria which have been discarded because they require the installation and usage of the apps, including criteria related to the functionality of the tools or the resulting user experience, such as feasibility, intuitiveness, learnability, efficiency, engagement, etc. The following paragraphs provide a brief explanation of the chosen characteristics with some examples. See Table 1-3 for complete lists of characteristics and their descriptions.

**Table 1:** Investigated characteristics of user documented food consumption data related to scientific relevance and extracted information (*n* = 176)

Characteristic	Description	Extracted information (n)
Dietary assessment method	The dietary assessment method used by the app for collecting food consumption data	Food diary (166), No information (8), Incidental food logging (2)
Food consumption inputs^a^	The type of food consumption data inputs supported	Generic input (91), Custom input (74), Labeled or packaged food products (44), Barcodes (scanned) (39), Water (30), Food images (21), Recipes (20), Restaurant dishes (19), Nutrient/Energy input (19), Diet plans (9), Voice input (4), Food log reminder (2), No information (2)
Precompiled food database	Whether the food consumption logging is supported by selecting foods from precompiled databases	Yes (93), No (83)
Food database compilation	The official food database the apps use for calculating nutrition and energy estimations	USDA (7)
User compiled databases^a^	The type of user compiled databases the app generates for logging references	Favorite eaten foods (29), Recently eaten foods input (15), Frequently eaten foods (14)
Nutrient/Energy estimation^a^	The unit or level of detail nutrient and energy consumption is estimated	Calorie (94), Macronutrients (78), Carbohydrates (49), Protein (49), Food score (26), Micronutrients (25), No information (20)
Portion size	Whether the app collect portion size estimations	Yes (96), No information (57), No (23)
Method portion size^a^	The methods that was used to collect portion size estimations	Standard serving sizes (59), Weight estimation (26), Volume estimation (9), Manual energy/nutrient input (5), Custom serving sizes (4)
Location	Whether the app collects information about where the consumptions took place	No (162), Yes (14)
Occasion	Whether the app collects information about the occasion or event of the consumptions	No (175), Yes (1)
Contextual data^a^	Data parameters the app collects about users other than food intake data	Motivation (107): Nutrition goals (59), Diet plans (38), Weight goals (32), Food preferences (29), Fitness goals (10), Fitness plan (10), Emotions (9), Health goals (7), Hydration goals (7), Stress level (5), Muscle building goals (3), Sleep goal (3), Health (108): Body weight (76), BMI (22), Medications (11), Symptoms (12), Body composition (11), Body measurements (9), Body image (8), Blood sugar (8), Blood pressure (8), Heart rate (7), BMR (7), Cholesterol (4), Physical fitness (4), Oxygen saturation (2) Physical activity (90): Exercise (59), Activity type (29), Steps (19), Activity level (14), Sleep (13) Uncategorized (34): Posts (27), Notes (22), Comments (6), Lifelogging data (3)
Interventional influences type^a^	The type of interventional influences the app contains that might have an direct influence on the recorded food intake behavior	Reminders/Notifications (54), Advices (53), Social support (23), Connected users (21), Coaching (19), Challenges (17), Personal feedback (14), Rewards (6), Encouragements (6), Allowance badge (4)
Sensors type^a^	The type of own external devices the app supports (exclusive devices of third party partner apps or health and fitness sensors)	Pedometer (4), Heart rate monitor (3), Accelerometer (3)
Third party health and fitness trackers^a^	The third party health and fitness trackers the app connects to	Fitbit (19), UP® – Smart Coach for Health (10), Health Mate - Steps tracker & Life coach (10), Misfit (6), Garmin Connect™ Mobile (4), Record by Under Armour, connects with UA HealthBox (2), Samsung Gear (1)
Aggregators^a^	The third party data aggregators the app connects to	HealthKit (31), GoogleFit (17), Healthgraph (5), S Health (5), Human Api (3), Validic (2), Fitnesssyncer (2), HealthVault (1)

^a^Per characteristic multiple inputs were possible and hence the individual percentages do not add up to 100%

**Table 2:** Investigated characteristics of user documented food consumption data related to data management and extracted information (n = 176)

Characteristic	Description	Extracted information (n)
Data export	Whether the data collected by the app is exportable directly via the apps infrastructure (not via integrated aggregators)	No information (117), Yes (55), No (4)
Access method^a^	The type of data export	File download (40), Email export (9), API (5), SDK (3), No information (3), Dropbox^b^ (3), AirDrop^c^ (1), Google Account^d^ (1), Google Drive^e^ (1)
Data format^a^	The format the data can be exported	PDF (18), CSV (18), Excel (9), No information (8), JSON (4), HTML (3), SQLite^f^ data file (2)
External data sources^a^	What type of third parties systems does the app exchange data with	Aggregators (44), Partner apps (40), Health and fitness trackers (24)

^a^Per characteristic multiple inputs were possible and hence the individual percentages do not add up to 100%

^b^cloud storage provider or online backup service that is also used as a file-sharing platform

^c^lets Mac and iOS devices share files wirelessly

^d^required for access to certain Google online services and supports app data storage

^e^personal cloud storage service that lets users store and synchronize digital content across computers, laptops and mobile devices

^f^SQLite is a relational database management system

*API* Application Programming Interface, *SDK*, Software Development Kit, *PDF* Portable Document Format, *CSV* Comma Separated Values, *JSON* JavaScript Object Notation, *HTML* Hypertext Markup Language

**Table 3:** Investigated characteristics of user documented food consumption data related to legal governance and extracted information (n = 176)

Characteristic	Description	Extracted information (n)
Website	Whether the app can be associated with a working home/support page	Yes (140), No (36)
Contact information	Whether the app vendor provides contact information	Yes (117), No (59)
Terms & conditions	Whether the app provides a terms of use document	Yes (69), No (107)
Privacy statement	Whether the app provides a privacy policy document	Yes (80), No (96)
Ownership^a^	The parties who hold the ownership of the user generated data (User content)	User (50), Vendor (1), No information (125)
Usage license vendor	Whether the app vendor retains the right to access and exploit the user generated data (publish, distribute, publicly display)	Yes (43), No (9), No Information (124)
Personally identifiable information collection	Whether the app collects personal identifiable information (e.g., during registration)	Yes (74), No (5), No Information (97)
Type personally identifiable information^a^	The types of personal identifiable information does the app collect	Email address (44), Name (37), Username and or password (28), Date of birth (18), Phone number (16), Registration (16), Health data (15), Address (14), Financial information (11), Gender (10), Additional data (8), Optional registration (8), Physical characteristics (7), Demographics (7), Mandatory registration (7), Image (5), Postcode (4), Location (3), No information (3), Interactions (1), Home address (1), Personal video (1), Social network handle (1), Ethnicity (1)
Public profile	Whether the app creates a public profile of the users personal data	Yes (38), No (5), No Information (133)
Privacy settings public profile	Whether the is user able to configure the privacy settings for his or her public profile	Yes (21), No (1), No Information (16)
Cookies	Whether the homepage/website of the app stores cookies on a user’s computer	Yes (61), No Information (115)
Web Beacons	Whether the homepage/website of the app stores web beacons on a user’s computer	Yes (25), No (2), No Information (149)
PII data sharing affiliates	Whether the collected personal identifiable data will be shared with affiliated third parties (confidentiality agreements)	Yes (51), No (8), With consent (4), No information (113)
PII data sharing non affiliates	Whether the collected personal identifiable data will be shared with unaffiliated third parties (without confidentiality agreements)	Yes (4), No (11), With consent (29), No information (132)
Usage Analytics	Whether the homepage/website of the app uses third-parties for advertising and usage analytics	Yes (41), No (1), No Information (134)
Data Storage^a^	The location where the system stores the data it collects	Device storage (78), Server storage (48), No information (81)
Data encryption	Whether the collected data is stored or transmitted in encrypted form	Storage: No information (176) Transfer: Yes (15), No information (161)
Data deletion	Whether the user is able to delete or ask for deletion of his or her personal identifiable information (e.g., after account termination)	Yes (33), No (1), No Information (142)

^a^Per characteristic multiple inputs were possible and hence the individual percentages do not add up to 100%

#### Scientific relevance characteristics

This was defined as how well the collected data meets the needs and standards of researchers in terms of the concepts measured [27]. The information collected reflected the methods and standards used for dietary intake assessments and the estimations of habitual food intake behaviours [28]. Information extraction properties included implemented methods for collecting food intake data, types of food data collected (e.g., generic foods, labeled products, images), and estimations of portion sizes and nutrient values. Information related to the collection of contextual data (e.g., activity, health, sleep) was collected as it offers the potential to better understand the determinants of food consumption behaviours [29]. Scientific relevance does not refer to testing the reliability and validity of the collected dietary assessment data. Rather by investigating these characteristics of the apps we aimed at getting indications about the potential usefulness of the data they generate for investigating habitual food intake and its determinants.

#### Data management characteristics

The FAIR data principles act as an international guideline for enhancing the ability to find, access and use scholarly data. FAIR stands for ‘Findable, Accessible, Interoperable, and Reusable’. In the present research we focused mainly on data access and data interoperability characteristics, including methods for data export, exchanged data formats and references to other relevant data.

#### Legal and ethical governance characteristics

These characteristics were based on some of the existing literature on the legal and ethical issues related to data collected by commercial mobile health apps [30–35] and ethics of secondary data analysis and big data [19, 23, 36]. We included criteria such as data ownership, data sharing, data usage, personally identifiable information, privacy and informed consent.

### Data collection

A web-based data collection tool was built using the open source Nodejs content management system Keystonejs (version 0.3.17) as an application framework. The tool consisted of a set of branched web forms for data input and data editing. The content and structure of the web form were based on the data characteristics defined for collecting information from the defined sources. The web form implemented various answering formats (widgets) including open format text and number input fields, as well as closed format input fields with predefined and selectable answering options. The tool was designed to allow for the management of these closed format input options and their definitions (except for the yes-no format). This had the advantage of providing the flexibility needed for explorative data collection, while at the same time applying a certain degree of standardization by making previously provided inputs and their definitions reusable. The tool also supports the visualization of app relevant information sources (e.g., screenshots, app descriptions, etc.) and for aggregations and visualizations of the extracted information. All collected information from app stores and online resources contained in the database have been exported and imported into an Excel file (see Additional file 1).

## Results

### The app sample

Most apps (90 and 91%) were listed in the category “Health and Fitness” in their respective app stores. The purpose of the majority of apps was to support some form of behavioural change, with weight management being the most commonly stated purpose. Since we selected apps based on mean user ratings (on a 5-point rating scale), user ratings of included apps were high, with a mean of *M* = 3.8 (*SD* = 0.7) for IOS apps and *M* = 4.0 (*SD* = 0.4) for Android apps. In 70% of the cases, apps included from the iTunes store were free of charge with the remaining paid apps ranging in price from £0.79 to £3.99. Apps included from the Android store were, in 87% of the cases, free of charge and the paid apps ranged in price from £0.55 to £7.61. Additional paid services or in app purchases were offered by 46% of all apps. The Android platform was supported by 88% of apps in our sample. IOS devices were supported by 109 apps (63%). Apps which also supported Windows and Blackberry devices accounted for 2% of our sample. Only 1 of the apps, the mySugr Diabetes Diary, was registered as a medical device as defined by the quality regulations and standards associated with that status [37]. In addition to monitoring blood glucose levels, this app supported the monitoring of daily carbohydrate intakes. In 80% of the cases, a website was available, which allowed for further investigation of the apps publicly available information. The websites of 4 of the apps were not available in English. Except for information extracted from the app stores, no further information was extracted from these websites. In 11% of the cases, no Uniform Resource Locator (URL) was provided, and no app associated home page was found on Google Search (a support URL is required for publishing apps in the iTunes store). In 8% of the cases an URL was provided, but the website was unavailable, and in 3% of the cases the address referred to a social media landing page. In cases where no website was available for an app, no further information, other than the information published in the apps stores, was investigated.

### Scientific relevance

#### Dietary assessment method

The most widely implemented method was a food diary (*n* = 166, 94%; see Table 1). Food diaries allowed for daily records of the foods and/or drinks people consumed at the individual level and at a certain moment in time (e.g., meals, snacks, date, time). Although in their feature descriptions 4% (*n* = 8) of the apps claimed to record food intake, no specifications could be obtained in the publicly available information regarding the specific method implemented to do so. A food image collection method for occasional photographic remembering and experience sharing purposes was implemented by 2 (1%) apps.

#### Dietary assessment inputs

Ninety-three (53%) apps allowed for inputs from pre-compiled food databases and 74 (42%) apps allowed for custom user compiled inputs. Links to verified sources of the precompiled database (e.g., Composition of foods integrated dataset; CoFID) were available for 7 (4%) apps. Food diaries allowed for various types of inputs. Generic food items could be logged in 91 (52%) apps. Labeled or packaged food products have been identified as possible input type in 44 (25%) apps and 39 (22%) apps implemented a barcode scanner for identification and logging of these labeled products. Food images have been allowed as input in 21 (12%) of the cases and recipes in 20 (11%). Some apps allowed for specific types of customizable or user-documented data inputs such as favorites (29; 16%), frequently consumed foods (14; 8%) or recently consumed foods (15; 8%).

#### Nutrient estimation

Based on the foods eaten, energy (94; 53%), macronutrients (78; 44%), and micronutrients (25; 14%) were estimated. In 8 (5%) of the apps, food images were used to estimate energy and nutrient intakes or provide a normative evaluation of the foods depicted in the images. These estimations or evaluations were provided by either diet coaches or users themselves. Three tools claiming to use an image recognition software were identified.

#### Portion size estimations

Portion size estimations were reported to be supported by standard household measures such as cups, spoons, slices (59; 34%), weight and volume (35; 20%), or visual aids in the form of images or graphics (1 app). No information on portion size estimation was provided for the remaining 46% of apps.

#### Interventional influences

One-hundred (57%) apps included some form of intended interventional influence on users’ food consumption behaviour, including nutrition advice (53; 30%), reminders and recommendations (54; 30%) in the form of eating and drinking reminders, notifications, badges or rewards for coming close to and reaching predefined weight or nutrition goals. Sources of social support and motivation including connected users following each other’s progress and posts (23; 13%), personal coaching for the achievement of user-specific diet or weight goals (19; 11%) and the option for inviting other users to compete or take part in weight loss or exercise challenges (17; 10%), were also identified.

### Contextual data

One-hundred and seven (61%) of the dietary assessment tools collected some form of data related to motivation, including users’ goals related to their desired intake of energy, nutrients, or water (59; 34%) or desired body weights (32; 18%) and states of physical fitness (10; 6%). Users’ preferences such as preferred foods were identified in 29 (16%) of the apps, and 9 (5%) apps allowed users to record their mood or emotions.

Health and physical fitness indicators were identified in 108 (61%) apps. These indicators included body weight (76; 43%), body mass index (22; 13%), or body composition (11; 6%). Symptoms, in the form of subjective evidence of current diseases, were found in 12 (6%) apps and records of drugs or other substances used to treat diseases or injuries in 11 (7%). Some apps allowed for monitoring of blood sugar (8; 5%), blood pressure (8; 5%) or blood oxygen saturation (2; 1%).

Contextual data related to users’ physical activity have been identified in 90 (51%) apps***.*** This includes various types of activities (29; 16%; e.g., swimming, cycling, running) and number of steps taken (19; 11%). Sleep and sleeping patterns have been identified in 13 (7%) apps. Twenty-seven (12%) of the apps offered social media platform features for exchanging data and information with other connected users. Thirteen (7%) of the apps allowed their users to share their data and progress updates with popular social media networks. Eleven percent of the tools in the sample were identified as allowing for inputs of dishes from restaurant menus. This implies that food consumption data collected by these tools might contain information regarding the location where the food was purchased. Geo-coordinates provided by a GPS unit were identified in one of the apps.

### Data management

In 55 (31%) apps the possibility for exporting user-documented food consumption data (from the app infrastructure, e.g., website) was identified (see Table 2). The most frequently implemented data export method was file download (40; 23%), which allowed users to download the collected data, in the form of a data file, directly from the apps’ websites. Some apps allowed for email export from within the app (9; 5%), whereby the exported data file is sent as an attachment to an email address specified by the user. Exported data was found to be in various standard formats including portable document format (PDF; 18; 10%), and comma-separated values (CSV; 18; 10%). Only a few app vendors allowed data export through a public API (Application Programming Interface; 5; 3%). APIs enable a more seamless distribution of data, in comparison to manual data file export. By allowing the sharing of data between authorized organizations and their IT systems (e.g., apps), processes can be automated without the need for manual intervention. All implemented APIs stated their ability to respond in the JavaScript Object Notation (JSON), which is a lightweight and widely recognized and supported open-standard data format [38].

Although only a few app vendors stated that they utilized a public API, about a quarter (40; 23%) of the apps exchanged data with at least one other dietary assessment tool included in the sample. Apps with the greatest number of connections with other dietary assessment apps in our sample were apps which implemented an API for data exchange such as Fitbit, connecting with 19 (11%) of the sampled apps, followed by Jawbone Up, and MyFitnessPal connecting with 10 (6%) and 3 (2%) of the sampled apps respectively. Twenty-four (14%) of the investigated dietary assessment apps connected to at least one popular health and fitness tracker (e.g., Garmin, Misfit, Withings), all which implemented an API for data access.

About a quarter (44; 25%) of the apps were exchanging data with at least one data aggregator or central data collection hub. Aggregators are designed to allow health and fitness apps to work together and collate their data. These various streams of data from apps and devices such as data on body weight, exercises, activities or dietary consumption can then be accessed and visualized on a single dashboard. We found in total twelve data aggregators which integrated with at least one of the diet apps in our sample. The aggregators which integrated with most apps in our sample were Apple’s HealthKit (31; 18%) and Google Fit (17; 10%). Other aggregators connecting to various diet apps in our sample were S-Health (5; 3%) HealthGraph (5; 3%) Human API (3; 2%) and Validic (2; 1%). All aggregators implement a documented API for data access.

### Legal and ethical governance

Sixty-nine (39%) apps in our sample provided a terms and conditions document, and eighty (45%) provided a privacy statement (see Table 3). In fifty (28%) apps the user was described as the owner of the data and in forty-three (24%) of the apps users were required to grant the app vendor an irrevocable, worldwide, and royalty-free license to commercially exploit the collected user-documented data including reproduction, adaptation, distribution, and publication. More specific data usage and exploitation purposes were not further described. Seventy-four (42%) apps were described as collecting personally identifiable information (PII) including name, email address, phone number or date of birth. Thirty-eight (22%) apps created a public profile of the collected data, and twenty-one (12%) stated that they offer privacy settings in order to restrict data publishing of those public user profiles. Fifty-one (29%) apps stated that vendors would share collected PII with other affiliated parties without written consent. These affiliated third parties are required to comply with the privacy policies of the app vendors. Sharing PII with unaffiliated parties, which are not bound by the privacy policies of the app vendors was described in twenty-nine (16%) of the cases. In those cases, vendors claimed to ask for written consent prior to data sharing.

## Discussion

We identified several potentials and challenges associated with the use of food consumption data collected by users of publicly available nutrition apps. Our findings have important implications for the scientific, technical, legal and ethical aspects associated with user-documented data and ultimately for developing strategies and policies aimed at the collection, integration, and sharing of this type of data.

### Scientific implications

In our app sample, for the most part, food diaries were used for assessing people’s diets, which is in line with previous findings [1, 2]. In addition to recording the time of consumption, food diaries have several advantages over other dietary assessment methods such as allowing for the collection of detailed actual intake data and being less prone to memory bias due to the focus on current food intake. Important challenges related to food diaries, however, are underreporting due to the large respondent burden and reactivity, meaning that keeping the diary affects habitual consumption behaviour. Hence, the completeness and quality of the user-documented food consumption data may decline over time [7]. Technical advances to support food logging might lower the user burden. Food diaries in our sample often allowed for precompiled food databases, which has been suggested to enable a more efficient and user-friendly data collection process [1, 2, 7, 8]. Barcode scanners which were also commonly described in our sample have been claimed to reduce the burden of food recording [14, 39] and have been evaluated as comprehensive, easy to use and non-intrusive data collection tools [40]. Similarly, food image processing technology, which has been characterized in some of the apps, aims at further increasing the accuracy of self-reported dietary recordings by increasing user adherence, automation, and standardization [41]. Although technical advances in dietary self-monitoring can make dietary assessment more efficient, they might not be sufficient for increasing users’ adherence to diet apps. Self-regulation techniques such as goal-setting and intention formation features were also identified in a large number of apps. These features might be needed to promote user engagement and sustained use of apps [17]. Finally, since a large number of apps allowed users to add new foods to the underlying databases for current and future references, a more complete and representative insight into the prevalence and day-to-day variability of individual preferences and dietary patterns might be supported.

Technical advances, however, come with challenges. The clear challenge for database-driven food logging approaches is the quality of the databases [39]. References to verified database compilation standards were only available for a small number of apps and tests of the quality of food databases underlying food consumption apps including estimated nutrients are nearly absent [6, 42]. In addition, the investigated apps provided a limited set of nutrients for the foods eaten, with the vast majority of apps focusing on intake of energy and macronutrients. Since very limited information was provided on the content of the food composition databases underlying the apps, their quality and the availability of data for specific nutrients and also the degree of missing data for the nutrients included is not known. This might be a barrier for research interested in the associations between specific nutrients and health outcomes. The investigated apps might be useful for assessing intake on the food level. Product brand names and recipes can provide valuable information for researchers interested in users’ food preferences and choices. Nutrient values for the consumed foods could be estimated post hoc by matching the identified foods to food entries in a quality controlled food composition database.

In addition, there is a clear scientific challenge to better understand the effects of the various app features on changes in food consumption behaviours [4, 10]. Food diaries are prone to changes in food consumption behaviours [43] and the presence of behaviour change features might often be non-evidence based [15, 44, 45]. This is problematic for generating unbiased insights into habitual food intake and its behavioural determinants and it complicates validation of apps against a reference standard [6].

Goal setting and intention formation features might not only increase user engagement but at the same time provide motivational and situational context to the collected food consumption data. Psychological factors such as users’ goals, preferences and habits were the most prominent in the apps and provide potentially relevant determinants of people’s food consumption behaviours [46]. Because energy expenditure drives food consumption [47], the collected data on body size and physical activity, including peoples’ routines, might additionally provide relevant physical and physiological context. Overall, though, the emphasis of contextual data was on parameters relevant for weight management including weight goals, energy expenditure, body weight or BMI. There are potential gaps in relevant determinants of food consumption such as data related to users’ emotional states, or the physical location of food consumption. Since the primary focus of the investigated tools was collecting data about the individual, relevant determinants of food consumption within the social and cultural context might have been lacking.

There seems to be a clear tendency towards integrating and enriching user-documented data with data from third-party apps or data aggregators [48, 49]. Food consumption episodes can be enriched with GPS locations, social media interactions or various kinds of health and activity related data through their connections with partner apps or aggregators. The strive towards integration and interconnectedness with other services and apps has the potential to provide a more complete overall summary about the user [44] and ultimately a more thorough understanding of the determinants of food consumption behaviours.

### Data management implications

The lack of procedures for exporting collected food consumption data in the investigated apps poses a barrier towards data sharing and analysis [50–52] and is considered an important criterion of data quality [27]. A significant challenge for self-tracking technologies is fragmented data scattered across multiple platforms [53, 54]. This is not only challenging for researchers interested in investigating this type of data but also forms an important barrier for motivating users to adopt and use self-tracking technologies. Since the majority of popular health apps do not seem to allow individuals access to their data beyond what is presented through the commercial interfaces, the evolving data integration and sharing platforms might also have important implications for data management practices. The emerging network of apps and data aggregators might provide an alternative and more efficient opportunity for data access. All sampled data aggregators implemented APIs for requesting and digesting food and related health data from various systems. In order to support processing and visualization of collected data irrespective of the source, these data platforms might provide some degree of initial harmonization of the data. Due to the potential lack of portability of data from one system to another [55, 56], accessing data entities already integrated from multiple data sources could be a very effective strategy from a data management perspective. Since data generated by external sources remains difficult to harmonize, however, imposing post-hoc standardizations and controls might render data inconsistent with its original source and hence provides data portability at the expense of data quality [57].

### Legal and ethical implications

The lack of formal documentation regarding terms of usage, ownership, and privacy identified in the apps we investigated, increases the risk of compromising data integrity and forms an important challenge for legal and ethical governance of user-documented food consumption data. There is a requirement for apps to cover data ownership and data privacy in their licensing agreement, which the consumer accepts at the time of first use [30–32]. Users express a clear interest in being in control over their generated data [51] and compromises to data integrity can occur when researchers are not aware of existing data ownership policies and consequently fail to adhere to the rights, obligations, and expectations regarding data usage [24]. In addition, a key principle of research ethics is that participants must have all of the information that might reasonably influence their willingness to participate in a study, including its purpose, implications, risks, and measures taken to protect appropriate levels of anonymity, confidentiality, or de-identification [58]. Vendors were often granted an irrevocable and royalty-free license to commercially exploit the user-documented data, however, without specifying its usage and purpose. The overall lack of documentation regarding these core elements of informed consent poses a clear challenge to maintaining the ethical and legal integrity of the data.

The new General Data Protection Regulation (GDPR) aims to standardize privacy rules and the protection of personal data across the European Union [59]. Considering the vast amounts of lifestyle and health parameters collected by the sampled apps and exchanged in a rapidly increasing network of integrated systems, distinguishing personal data from non-personal or non-identifiable data might become challenging. Food consumption behaviour is deeply rooted in people’s personal and social identities [60, 61] and might make reference to locations, diseases, ethnical origins, or ethical and religious convictions. Hence in the context of the privacy standards set by the GDPR, it is prudent to argue that user-documented food consumption data could be categorized as personal data (or at least certain elements of it). This would have strong legal and ethical implications for future privacy policies regarding informed consent and sharing of these data, as well as important technical implication for data access and integration due to the “right of data portability” and the “right to be forgotten” associated with personal data. Legal and ethical government issues to some extent apply to all kinds of digital apps collecting personal identifying information. Since secondary data, however, varies in terms of the amount of identifying information it contains [19], the complex and dynamically increasing network of diet apps and its implications regarding personal data requires a more thorough investigation on a case by case basis.

### Limitations and future research

The extent to which information about the apps was publicly available was critical for collecting the relevant data about the apps in our sample. For a large number of apps, however, the relevant public sources of information such as homepages or privacy and terms of use statements were unavailable. The availability of information is considered an important indication of the quality of online products and services [62], and hence the lack of available information can be interpreted as a potential limitation to their utilization. We are aware that due to our chosen method of investigating publicly available information of apps, our sample might lack important information that has not been publicly provided or might even contain false or biased information based on misinterpretations by the authors or misleading publication and marketing strategies by app vendors. Since we did not download a random subset of apps in order to validate our interpretations, we are unable to provide estimations regarding the extend of these potential errors.

Considering the sheer number of potentially relevant apps that collect user-documented food consumption data, the fundamental challenge was to provide a selection of apps which was able to capture the variety of data collection apps available on the market. The app selection procedure was dependent on the order of the apps retrieved from the app stores. This strategy might be biased based on the estimated app relevance assigned by the app stores at that moment of time. In addition, the present selection of apps was limited to apps available in the UK storefronts. Although a large number of apps in our sample were also available in other countries, a selection of apps searched in app stores of different countries might have revealed a somewhat different set of apps. Finally, the present research did not aim to create an inventory of food consumption apps, since that would require a continuous research effort. Rather, we aimed to gather information from a representative sample of apps and learn from this highly dynamic market. Since the content in the apps stores is changing at a fast pace, the selection of apps for the present sample should be considered a snapshot of apps at a certain moment in time.

The present research did not aim to provide a general quality framework nor recommendations for researchers who seek to identify the scientific relevance of diet apps in general. The selection of characteristics indicative of scientific relevance needs to be tailored to the needs, quality criteria and protocols, specific to a particular research objective. That is, objectives on the assessment of dietary intake and health behaviour, effectiveness in weight or diabetes management, or understanding consumer behaviour at the individual or group level, emphasize different sets of quality characteristics which do or do not qualify an app as ‘fit for purpose’.

Our aim was not to evaluate the quality of user-documented data collected by individual apps or to provide informed recommendations for choosing one app over the other. In order to get app specific quality evaluations and informed comparisons of apps, we believe it is important to further examine and validate a smaller selection of apps more closely. In particular, the evaluation and validation of food databases are warranted. In a recent study, Maringer et al., investigated the quality of labelled food product databases underlying popular diet apps with barcode scanners [63]. The authors concluded that, due to the variations in availability and accuracy of nutrient information contained in these databases, they lack the necessary consistency and accuracy for assessing dietary intake on the nutrient level. In addition, Maringer et al., reported that for some apps, food consumption data were not available for individual foods or meals, but was aggregated over certain periods of time. Hence in order to get more app specific evaluations regarding the availability and accuracy of user-documented data, those apps which allow for the export of collected data in a standardized format needs closer inspection. In addition, data aggregators might help to overcome some of the fundamental challenges related to user documented food consumption data from apps, including data linkages to contextual data, seamless data access, data harmonization and standardization. Further research is needed, however, in order to better understand the types and quality of data they exchange.

## Conclusion

Considering the dynamically changing domain of publicly available food consumption apps and their increasing use of technical innovations, determining whether the data collected by users of these apps are “fit for purpose” involves a continuous research effort. User-documented food consumption data has the potential of providing scientifically relevant insights into the prevalence and variability of individual preferences and dietary patterns. The collection of lifestyle data and strive towards integration and interconnectedness of data might help to better understand the determinants of food consumption behaviours. An important challenge for an efficient data management strategy, however, seems to be the lack of available or documented data access. The identified interconnectedness of apps and their data provides new opportunities for data management (efficient data access, standardized formats, data linkage). The overall lack of documentation regarding terms of usage and data privacy poses a clear challenge for legal and ethical data integrity. The strive towards integration and interconnectedness of user-documented data makes this task even more challenging.

## Additional file

Additional file 1: Collected information from app stores and online resources. (XLSX 3032 kb)

## Abbreviations

API: Application Programming Interface
CSV: Comma Separated Values
EU: European Union
GDPR: General Data Protection Regulation
GPS: Global Positioning System
JSON: JavaScript Object Notation
PDF: Portable Document Format
PII: Personally Identifiable Information
RI: Research Infrastructure
UK: United Kingdom
URL: Uniform Resource Locator

## Footnotes

1 iTunes-search module version 1.0.1. Nodejs module to search app data on the iTunes search api. Url: https://github.com/connor/itunes-node

2 Google-play-scraper module version 0.2.1. Nodejs module to search app data on the Google Play store. Url: https://github.com/facundoolano/google-play-scraper
